# Supplementary material for: Highly Regioselective Synthesis of Bisadduct[C70] Additive toward the Enhanced Performance of Perovskite Solar Cells
Source: Nanomaterials (Basel). 2022 Jul 9;12(14):2355. doi: 10.3390/nano12142355 (PMC9324603; doi:10.3390/nano12142355)
Supplement: Supplementary file 1 [file nanomaterials-12-02355-s001.zip › nanomaterials-1747131-supplementary.pdf]

nanomaterials

MDPI

Supplementary Materials

Highly Regioselective Synthesis of Bisadduct[C_{70}] Additive toward the Enhanced Performance of Perovskite Solar Cells

Muqing Chen *, Yanyan Zeng, Gui Chen and Yongfu Qiu *

School of Environment and Civil Engineering, Dongguan University of Technology, Dongguan 523808, China; double124@126.com (Y.Z.); chengui@dgut.edu.cn (G.C.)
* Correspondence: mqchen@ustc.edu.cn (M.C.); qiuyf1979@foxmail.com (Y.Q.)

1. Experimental details

1.1. Theoretical calculation

DFT calculations were conducted with Gaussian 09 package [1] using the B3LYP functional with the 6-31G(d) basis set [2] for C, H, N atoms.

1.2. Materials

FTO-coated glass substrates with a sheet resistance of 13 ± 1.5 Ω/sq were purchased from NSG Group, Japan. CH_3NH_3I was bought from Xi'an Polymer Light Technology Corp, Xi'an, China. PbI_2 was obtained from Tokyo Chemical Industry Co., Ltd (Tokyo,Japan). Dimethylformamide (DMF), dimethylsulfoxide (DMSO), chlorobenzene, isopropanol, and acetonitrile were purchased from Sigma-Aldrich, Darmstadt, Germany. C_{60} was purchased from Suzhou Dade Carbon Nanotechnology Co. Ltd, Suzhou, China. All reactants and solvents were used as received without further purification. Sodium azide and 1, 2-bis(bromomethyl)benzene were purchased form Aladin (Shanghai, China).

2. Synthesis, separation and structure characterization

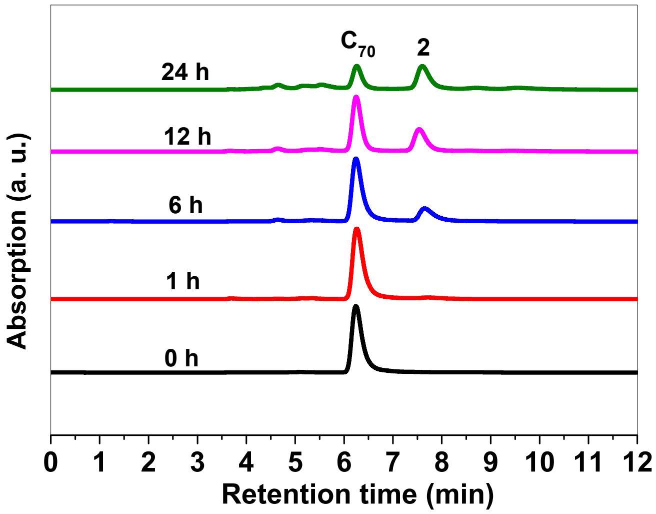

Figure S1. HPLC profiles of the reaction mixture of C_{70} with 1, 2-bis(azidomethyl)benzene monitored at different reaction times. Conditions: Buckyprep column (ø 6.4 mm ×250 mm), 0.8mLmin^{-1}, chlorobenzene flow, 330 nm detection wavelength.

Table S1. The crystal data of **2**.

Identification code	2
Empirical formula	$C_{159}H_{16}N_{12}O_{0.5}S_6$
Formula weight	2294.20
Temperature/K	100(2)
Crystal system	monoclinic
Space group	C2/c
a/Å	37.0550(16)
b/Å	13.6594(6)
c/Å	33.8788(15)
α/°	90
β/°	98.085(2)
γ/°	90
Volume/Å^3	16977.3(13)
Z	8
ρ_{calc} g/cm^3	1.795
μ/mm^{-1}	0.307
F(000)	9232.0
Crystal size/mm^3	0.090 × 0.070 × 0.030
Radiation	synchrotron (λ = 0.7749)
2Θ range for data collection/°	4.26 to 57.148
Index ranges	−45 ≤ h ≤ 45, -16 ≤ k ≤ 16, −41 ≤ l ≤ 41
Reflections collected	114881
Independent reflections	16701 [R_{int} = 0.0567, R_{sigma} = 0.0354]
Data/restraints/parameters	16701/0/1581
Goodness-of-fit on F^2	1.180
Final R indexes [I>=2σ (I)]	R_1 = 0.1085, wR_2 = 0.3177
Final R indexes [all data]	R_1 = 0.1289, wR_2 = 0.3416
Largest diff. peak/hole /e Å^{-3}	1.89/−3.10

3. Electrochemical measurements and theoretical calculation

Table S2. Redox potentials [a] of C_{70} and **2**.

Compound	$^{red}E_1$[V]	$^{red}E_2$[V]	$^{red}E_3$[V]	
C_{70}	−1.12	−1.50	−1.70	This work
Bis-adduct **2**	−1.14	−1.52	−1.92	This work
C_{70}	−1.05	−1.41	−1.83	3
C_{70}-bis-anthracene	−1.21	−1.53	−1.99	3

[a] Values obtained by CV in V vs.$F_c/F_c^{+\cdot}$

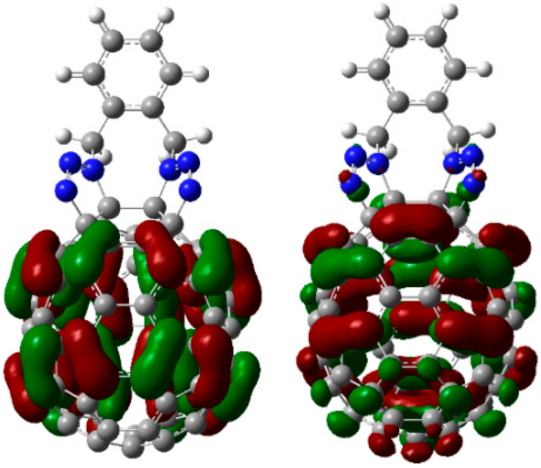

Figure S2. The electronic structures of **2** at HOMO and LUMO energy levels.

Theoretical calculation was employed to observe the change of electronic structure shown in Figure S2. After grafting the rigid phenyl tether onto C_{70}, electronic density distribution mainly locates on the surface of carbon cage under LUMO and HOMO energy level, demonstrating that there is no charge transfer between the functional group and carbon cage. The theoretical HOMO and LUMO energy levels are −5.93 eV and −3.38 eV, respectively.

4. Preparation and measurements of PSCs

4.1. Preparation of PSCs

Experimental section: ITO-coated glass substrates in the size of 1.5 cm × 1.5 cm were patterned by laser etching. Substrates were washed by ultrasonic cleaning and rinsing in the detergent aqueous solution, deionized water, acetone, and isopropanol, dried by clean air flow and finally treated via UV-O_3 for 15 min. The diluted SnO_2 colloidal solution (2.67 wt%) was spin-coated onto the ITO substrate at 3000 rpm for 30 s, then annealed in ambient air at 150 °C for 30 min. After the substrate was cooled down to the room temperature, thereafter it was treated by UV-O_3 for 3 min again for further deposition. The $MAPbI_3$ precursor was prepared by dissolving PbI_2 (1.1 M), MAI (1 M) into the mixed solvents of DMF: DMSO=7:3 (V: V) stirred overnight before using. The solution was spin-coated at 4000 rpm for 30 s and 100 μL of chlorobenzene (CB) was dropped onto the film at 10 s after the beginning of the program progress. Then the substrates were annealed at 100 °C for 10 min. For the preparation of perovskite layer doped additive **2**, a solution of **2** in CB with different concentrations was added into the perovskite precursor solution with volume ratio of 6:100 to obtain the corresponding weight ratios of additive **2**: PbI_2. The hole transport materials solution was prepared by dissolving Spiro-OMeTAD in CB (72.3 mg/m), with the additives containing 29 μL 4-tert-butyl pyridine, 17.5 μL Li-TFSI in acetonitrile (520 mg/mL) and 29 μL of FK209 in acetonitrile (300 mg/mL). Finally, 70 nm thickness of silver was thermal evaporated as top anode via a shadow mask. The effective area is 0.04 cm^2 defined by mask.

4.2. Measurements and Characterization of PSC devices

The current density−voltage (J−V) characterizations were conducted using a Keithley 2400 source measurement unit (Beaverton, OR, USA) under simulated AM 1.5 irradiation (100 mW cm^2) with a standard xenon lamp-based solar simulator (Oriel Sol 3A, CA, USA). The solar simulator illumination intensity was calibrated with a monocrystalline silicon reference cell (Oriel P/N 91150 V, with KG-5 visible color filter) calibrated by the National Renewable Energy Laboratory (NREL, WA, USA). The EQE measurements were carried

out on an Oriel Intelligent Quantum Efficiency (IQE, CA, USA) 200TM measurement system equipped with a tunable light source. XPS measurements were conducted on a Thermo ESCALAB 250 instrument (MA, USA) with a monochromatized Al K*α* X-ray source. SEM images were obtained using a field emission scanning electron microscope (FEI Quanta 200, OR, USA).

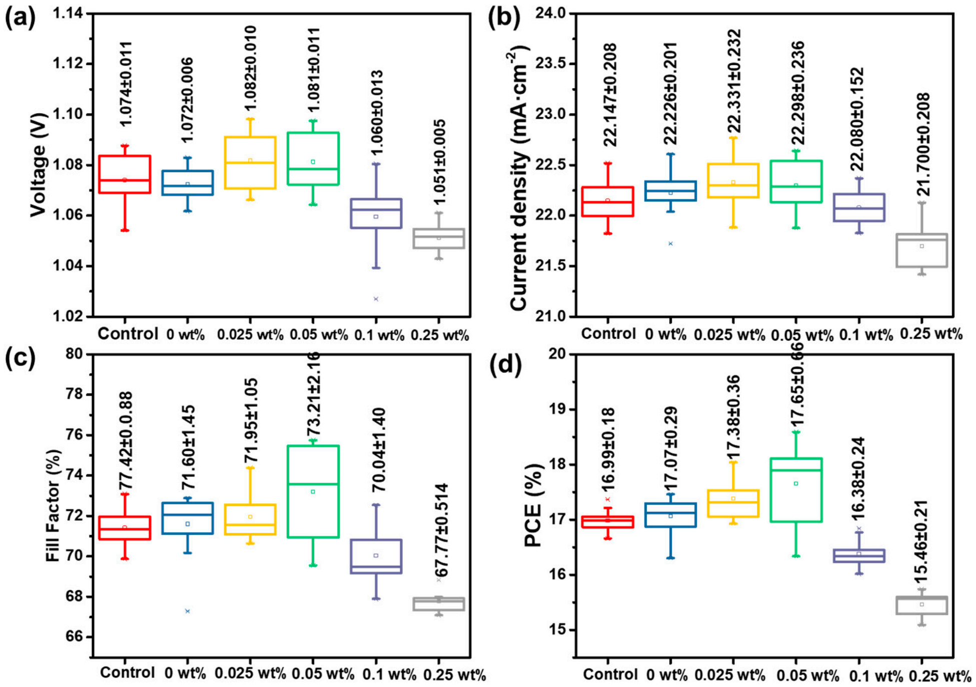

Figure S3. Box plots of V_{oc} (**a**), J_{sc} (**b**), FF (**c**) and PCE (**d**) for PSC devices with varying additive **2** concentration.

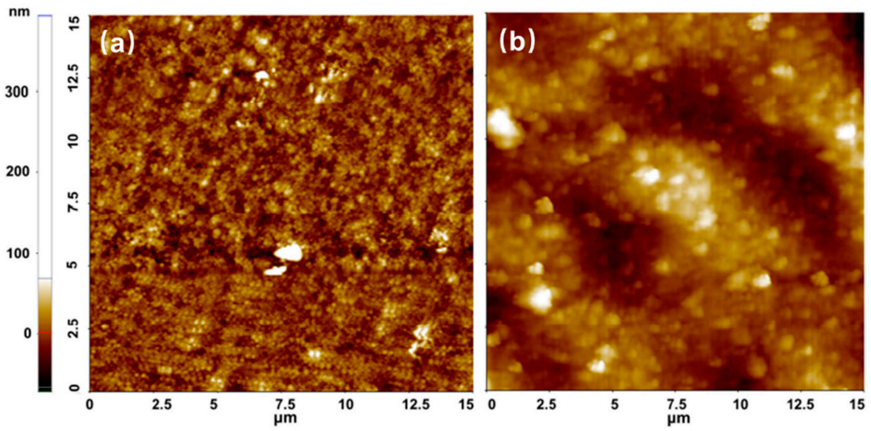

Figure S4. AFM images of $MAPbI_3$ perovskite film (**a**) and $MAPbI_3$/**2** film (**b**).

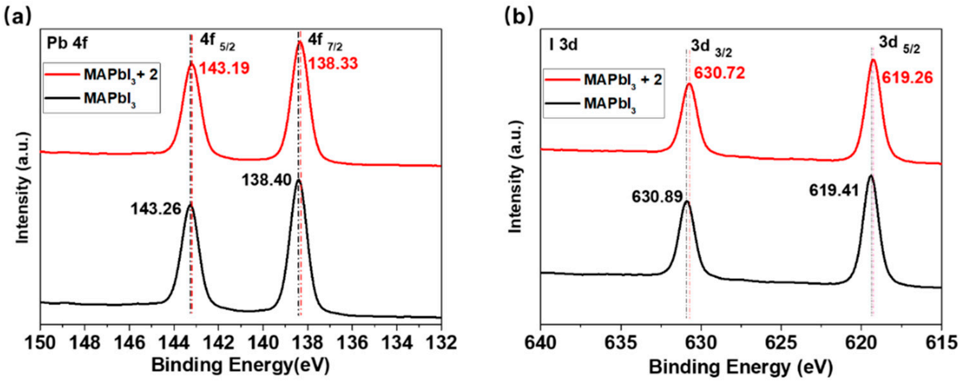

Figure S5. XPS images of Pb_{4f} and I_{3d} signals of $MAPbI_3$ perovskite and $MAPbI_3$/**2** films.

References

[1] Frisch, M.J.G.; Trucks, W.; Schlegel, H.B.; Scuseria, G.E.; Robb, M.A.; Cheeseman, J.R.; Scalmani, G.; Barone, V.; Mennucci, B.; Petersson, G.A.; et al., Gaussian 09, Gaussian, Inc., Wallingford CT, USA, 2009.

[2] Hehre, W.J.; Ditchfield, R.; Pople, J.A. Self—Consistent Molecular Orbital Methods. XII. Further Extensions of Gaussian—Type Basis Sets for Use in Molecular Orbital Studies of Organic Molecules. *J. Chem. Phys.* **1972**, *56*, 2257–2261.
